# Cardiovascular risk profile according to World Health Organization medical eligibility criteria for contraceptive use of women using hormonal contraception in Rio de Janeiro state, Brazil

**DOI:** 10.1002/ijgo.70548

**Published:** 2025-09-20

**Authors:** Isabela Pereira Fonseca Brusth, Juliana Montani Raimundo, Helene Nara Henriques Blanc

**Affiliations:** ^1^ Pathophysiology Laboratory Federal University of Rio de Janeiro — Multidisciplinary Center UFRJ Macaé Rio de Janeiro Brazil; ^2^ Research Group in Pharmacology of Bioactive Products Federal University of Rio de Janeiro — Multidisciplinary Center UFRJ Macaé Rio de Janeiro Brazil

**Keywords:** cardiovascular profile, hormonal contraception, women's health

## Abstract

**Objective:**

Hormonal contraception, especially combined oral contraceptives, is associated with an increase in cardiovascular risk. Thus, we aimed to analyze the use of hormonal contraceptive methods in the state of Rio de Janeiro (Brazil), with a focus on determining whether contraceptive use follows the cardiovascular profile of women according to the World Health Organization Medical Eligibility Criteria for Contraceptive Use (MEC).

**Methods:**

This was a descriptive cross‐sectional study based on a self‐completed online survey about information concerning socioeconomic status, cardiovascular profile, and hormonal contraceptive use. A total of 501 responses were evaluated.

**Results:**

Combined oral contraceptive was the choice of 58.97% of participants. Our data show that 88.27% of participants presented at least one cardiovascular risk factor, with sedentary lifestyle, overweight, and obesity being the most prevalent. Regarding cardiovascular risk factors considered in MEC, 81.60% of participants use the appropriate contraceptive for their health. Most women reported using the contraceptive with a prescription, including 83.33% of participants in categories 3 and 4, which represent contraindications for hormonal contraceptive use according to MEC.

**Conclusion:**

Although most women living in Rio de Janeiro use an appropriate hormonal contraceptive, clinical management and cardiovascular risk assessment must be improved to protect women's health and ensure an evidence‐based family planning strategy.

## 1 INTRODUCTION

Contraceptive methods are used by 966 million women worldwide. Of these, more than 247 million use hormonal contraception, an important component of family planning.^1^ Hormonal contraceptives include combined (estrogen plus progestogen) and isolated (progestogen‐only) formulations utilizing different routes of administration.^2^, ^3^ Additional benefits and also risks have been described for hormonal contraceptive methods. For example, in terms of benefits, combined oral contraceptives (COC) reduce the risk of pelvic inflammatory disease and developing ovarian and endometrial cancer, while progestogen‐only contraceptives (POC) can be used by breastfeeding women.^4^

Since hormonal contraceptives came into the market in 1960, the risk of cardiovascular disease (CVD), mostly thrombosis, has been monitored. Ethinyl estradiol, the main estrogen used in COC, has been shown to induce changes in the activity of coagulation factors, leading to a prothrombotic state.^5^ This effect can be increased by the progestogen component of COC, especially third‐generation progestogens.^6^, ^7^ COC is associated with an increased risk of venous thromboembolism, acute myocardial infarction, and stroke.^8^, ^9^ This risk is even higher for women with certain health diseases, such as diabetes, hypertension, obesity, and hyperlipidemia and a history of thromboembolism and heart disease.^10^, ^11^ Thus, POC is considered a better option for women with cardiovascular (CV) risk factors.^7^, ^12^

In this context, the World Health Organization (WHO) Medical Eligibility Criteria for Contraceptive Use (MEC)^10^ provides guidelines on the safety of different types of contraceptives based on specific medical conditions and characteristics, taking into account whether the benefits of the use outweigh the risks associated with it. This document is an important tool for family planning as hormonal contraception use is considered a women‐specific CV risk factor.^13^

Although CVD is the leading cause of women's mortality in Latin America^14^ and in Brazil,^15^ few studies have assessed the adequacy of hormonal contraceptives used by Brazilian women, and most studies have focused only on oral formulations users.^16^, ^17^ Thus, this study aimed to analyze the use of hormonal contraceptive methods in the state of Rio de Janeiro (Brazil), focusing on the assessment of whether contraceptive use is following the CV profile of women and the MEC.

## 2 METHODS

This is a descriptive cross‐sectional study based on a self‐completion electronic survey that occurred in Rio de Janeiro state, southeastern Brazil, which has approximately 16.46 million inhabitants.

The study was approved by the Human Research Ethics Committee at Centro Multidisciplinar UFRJ‐Macaé (Protocol no. 50584021.2.0000.5699) and followed the ethical guidelines outlined in Resolution 466/2012.

Participants were recruited via WhatsApp, Instagram, and Facebook from November 2021 to February 2022. For the online survey, snowball sampling was used. Women using a hormonal contraceptive method at the time of the survey, having access to the internet, aged 18–49 years, and living in Rio de Janeiro were included in the study. The exclusion criterion was pregnancy at the time of the survey.

The survey was designed using clear, accessible, and straightforward language, with additional explanations provided for technical terms to ensure full understanding of the questions. The contact information of the project coordinator was available in case of questions. Women were informed that their participation was voluntary and could be interrupted at any time.

The survey link was created using Google Forms, assessed only after electronic consent, and was composed of 32 questions about socioeconomic data (age, color, marital status, monthly family income, and educational level), CV profile, and contraceptive use.

The contraceptives were categorized according to the four categories of WHO MEC: (1) a condition for which there is no restriction for the use of the contraceptive method; (2) a condition where the advantages of using the method generally outweigh the theoretical or proven risks; (3) a condition where the theoretical or proven risks usually outweigh the advantages of using the method; and (4) a condition that represents an unacceptable health risk if the contraceptive method is used (WHO, 2015). Contraindications to hormonal contraceptive use, categories 3 and 4, were determined by the existence of at least one of the following conditions: hypertension, women over 35 years who currently smoke, thrombophilia, actual CVD, history of CVD, or family history of CVD.

**FIGURE 1:**
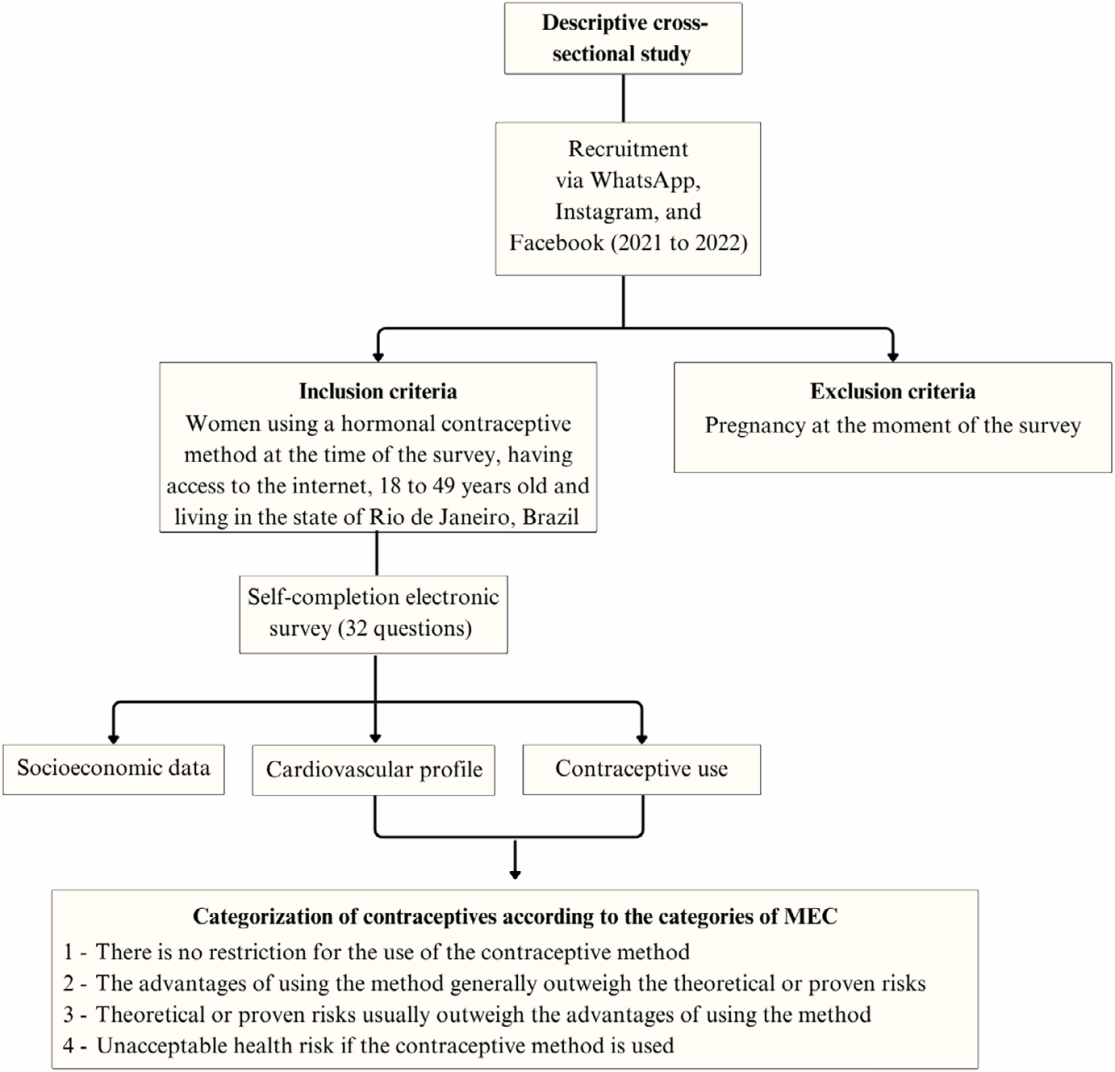
Methodological flowchart of the study design and data collection process.

The survey included questions on the following health criteria to classify the contraceptives according to WHO MEC: smoking (over 35 years old), obesity (weight and height were used to calculate body mass index), hypertension, high blood pressure during pregnancy, actual CVD, history of deep vein thrombosis/pulmonary embolism or current disease, ischemic heart disease, hyperlipidemia, diabetes and family history of thrombosis/pulmonary embolism. Figure 1 summarizes the study methodology.

The sample size (448) was calculated by using OpenEpi Calculator, considering the number of women of reproductive age living in Rio de Janeiro and using hormonal contraceptives, with a 97% confidence interval and a 5% margin of error.

For statistical analysis, the contraceptives were categorized into two groups: combined contraceptives (COC, combined injectable contraceptive, vaginal ring, and transdermal patch) and POC (progestogen‐only pills, levonorgestrel‐releasing intrauterine system—LNG‐IUS, progestogen‐only injectable contraceptive, and subdermal implant).

To assess the association between the type of contraceptive used (combined vs. POC) and demographic and clinical characteristics, the *χ* ^2^‐test of independence and Fisher's exact test were used. Statistically significant *χ* ^2^‐test or Fisher's exact test results were followed by an analysis of adjusted standardized residuals (Pearson residuals) to identify which categories showed observed frequencies that differed from expected values. Given the impact of sample size on *P*‐values, effect size measures were calculated for all tests (Cramér's V). The effect of CV risk factors on the type of contraceptive used (combined vs. POC) was also assessed using a binary logistic regression model. Age was included in the model as a control variable.

All statistical analyses were conducted using R software version 4.5.0 (R Core Team, 2025), with a significance level (α) set at 5%.

## 3 RESULTS

A total of 609 participants responded to the online survey, of which 107 responses were excluded because they did not match the inclusion criteria, yielding 501 participants. Most women aged 21–29 years (58.68%) were self‐declared white (56.09%), single (56.28%), with monthly family income between US$200 and US$800 (46.71%), and had completed high school (52.10%) (Table 1).

**TABLE 1:** Socioeconomic data of women using hormonal contraception in Rio de Janeiro.

Factor	No (%)
Age group (years)	
18–20	30 (5.99)
21–24	166 (33.13)
25–29	128 (25.55)
30–34	72 (14.37)
35–39	55 (10.98)
40–44	37 (7.38)
45–49	13 (2.59)
Color	
White	281 (56.09)
Brown	135 (26.95)
Black	75 (14.97)
Asian	7 (1.40)
Indigenous	0 (0)
Rather not answer	3 (0.60)
Marital status	
Single	282 (56.28)
Married or in a common‐law marriage	199 (39.72)
Divorced	15 (2.99)
Widow	3 (0.60)
Rather not answer	2 (0.40)
Monthly family income (USD)	
<213	71 (14.17)
214–854	234 (46.71)
855–2563	130 (25.95)
>2563	33 (6.59)
Rather not answer	33 (6.59)
Education level	
Incomplete elementary school	3 (0.60)
Elementary school	2 (0.40)
Incomplete high school	12 (2.39)
High school	107 (21.36)
Incomplete undergraduate	154 (30.74)
Undergraduate	92 (18.36)
Incomplete graduation	32 (6.39)
Graduation	98 (19.56)
Rather not answer	1 (0.20)

Our data indicated that 88.27% of participants have at least one CV risk factor, and only 6.36% of participants reported actual CVD. Among the CV risk factors investigated, sedentarism, overweight, and obesity were the most prevalent. In addition, 47.51% of women reported a family history of CVD and 25.24% of women who had already been pregnant reported gestational hypertension.

Combined contraceptives were the contraceptive choice of 58.97% of participants, followed by progestogen‐only pills (13.74%) and LNG‐IUS (12.35%). Injectable contraceptives (combined and progestogen‐only) were used by 10.56% of women, and only 4.38% reported the use of a subdermal implant, vaginal ring, or transdermal patch. Of participants, 69.78% reported using the contraceptive method for at least 1 year, with 29.63% using it for more than 5 years.

Table 2 shows the use of combined contraceptives and POC according to socioeconomic status. Data analysis showed that there was an association between age and the type of contraceptive used (*χ* ^2^(3) = 27.115; *P* < 0.001; Cramer's *V* = 0.233). The use of POC was less frequent among younger women (up to 29 years old) and more frequent in women aged 40–49 years. In addition, there was an association between marital status and the type of contraceptive used (*χ* ^2^(4) = 27.435; *P* < 0.001; Cramer's *V* = 0.234). The use of POC was more frequent in married/common‐law marriages and widows and less frequent in single women.

**TABLE 2:** Contraceptive use according to socioeconomic status in Rio de Janeiro.

Socioeconomic characteristics	Combined contraceptives (*n* = 336)	Progestogen‐only contraceptives (*n* = 165)	*P*	ES
Age group (years)			<0.001^a^	−0.208
<20	24 (7.1)	6 (3.6)		
20–29	214 (63.7)	80 (48.5)		
30–39	79 (23.5)	48 (29.1)		
40–49	19 (5.7)	31 (18.8)		
Color			0.362^b^	0.085
White	184 (55.1)	97 (59.1)		
Brown	88 (26.3)	47 (28.7)		
Black	56 (16.8)	19 (11.6)		
Asian	6 (1.8)	1 (0.6)		
Indigenous	2 (—)	1 (—)		
Monthly family income (USD)			0.091^a^	−0.078
<213	48 (15.3)	23 (14.8)		
214–854	166 (52.9)	69 (44.5)		
855–2563	83 (26.4)	47 (30.3)		
>2563	17 (5.4)	16 (10.3)		
Rather not answer	22 (—)	10 (—)		
Marital status			<0.001^b^	0.233
Single	116 (34.6)^c^	83 (50.6)^c^		
Married or in a common‐law marriage	6 (1.8)^c^	9 (5.5)^c^		
Divorced	213 (63.6)^c^	69 (42.1)^c^		
Widow	0 (0)^c^	3 (1.8)^c^		
Rather not answer	1 (—)^c^	1 (—)^c^		
Education level			0.115^a^	−0.071
Incomplete elementary school	3 (0.9)	0 (0)		
Elementary school	1 (0.3)	1 (0.6)		
Incomplete high school	10 (3)	2 (1.2)		
High school	72 (21.5)	35 (21.2)		
Incomplete undergraduate	108 (32.2)	46 (27.9)		
Undergraduate	58 (17.3)	33 (20)		
Incomplete graduation	25 (7.5)	7 (4.2)		
Graduation	58 (17.3)	41 (24.8)		
Rather not answer	1 (—)			

*Note*: Brazilian minimum wage BRL 1.100.

Abbreviation: ES, effect size; —, Not calculated.

^a^ *χ* ^2^‐test of independence.

^b^ Fisher's exact test.

^c^ Indicates cells in which the expected values differ statistically from the observed values.

According to the responses about the CV risk factors and the contraceptive method used, we investigated whether the contraceptive used was following WHO recommendations.^10^ We found that 57.68% of women do not have CV risk factors included in the WHO criteria. Then, the remaining 42.31% (212) were categorized. Of these, 81.60% of participants use the recommended contraceptive for their health condition (WHO category 1, 12.26%; and category 2, 69.34%), while 18.40% use it incorrectly, with 16.98% in category 3 and 1.42% in category 4. The most common risk factors in categorization 3 were obesity (22 women) and hypertension (21 women). In category 4, two out of three women had thrombophilia.

Regarding combined contraceptives, different CV risk factors present on the WHO MEC criteria were identified among the users of all methods. The transdermal patch users showed a family history of CVD and smoking (25% for each CV risk factor). For vaginal ring users, obesity, hypertension, history of gestational hypertension, and smoke were observed (12.5% for each CV risk factor). Among users of combined injectable contraceptives, the most frequently observed CV risk factor was obesity (27.6%), followed by hypertension (6.9%) and CVD (3.5%). In addition to these, gestational hypertension and smoking were each reported by 13.8% of women, and a family history of CVD was identified in 10.3% of users. In the group using COC, obesity was the most prevalent risk factor (20.3%). A family history of CVD was reported by 10.0%, hypertension by 6.2%, and gestational hypertension was present in 5.2% of users. Smoking (1%), CVD (1%), and thrombophilia (0.7%) were less common.

For women using progestin‐only injectable contraceptives, obesity and gestational hypertension were the most common risk factors (each found 25.0%). Hypertension was observed in 8.3%, smoking in 4.2%, and a family history of CVD in 4.2%. Among users of LNG‐IUS, the CV risk factors related were gestational hypertension (11.5%), obesity (9.8%), family history of CVD (6.6%), thrombophilia (4.9%), hypertension (3.3%), and a personal history of thrombosis (1.6%). Among users of POC, 26.5% were obese, and both hypertension and gestational hypertension were observed in 11.8% of cases. Smoking was reported by 1.5% and 5.9% had a family history of CVD.

Table 3 shows the association between the type of contraceptive, combined or POC, and CV risk. We observed that the use of POC was more frequent among women who reported not having high cholesterol and less frequent among those who reported using medication to control cholesterol levels (Table 3). As observed in the hypothesis tests, the binary logistic regression model indicated that the only CV risk factor associated with the type of contraceptive used is elevated cholesterol. Women with high cholesterol who use medication for its control have a statistically lower likelihood (odds ratio <1) of using POC compared to women who reported not having high cholesterol (Table 4).

**TABLE 3:** Association between contraceptive formulation and cardiovascular risk factors of women in Rio de Janeiro.

Factor	Combined contraceptives (*n* = 336)	Progestogen‐only contraceptives (*n* = 165)	*P*	ES
BMI			0.207^a^	−0.056
Underweight	14 (4.2)	3 (1.8)		
Healthy weight	164 (49.1)	72 (43.6)		
Overweight	88 (26.3)	59 (35.8)		
Obesity	68 (20.4)	31 (18.8)		
Inconclusive answer	2 (—)			
Diabetes			0.610^b^	0.065
Non‐diabetic	317 (94.6)	155 (93.9)		
Did not know	13 (3.9)	6 (3.6)		
Type 1	0 (0.0)	1 (0.6)		
Type 2	5 (1.5)	3 (1.8)		
Rather not answer	1 (—)	0 (0.00)		
Sedentarism^a^			0.491^c^	0.031
No	155 (46.1)	70 (42.4)		
Yes	181 (53.9)	95 (57.6)		
High cholesterol			0.039^c^	0.129
No	246 (73.2)^d^	136 (82.4)^d^		
Did not know	34 (10.1)	10 (6.1)		
Yes. not medicated	47 (14.0)^d^	12 (7.3)^d^		
Yes. medicated	9 (2.7)	7 (4.2)		
Smoking			0.457^a^	0.034
No	308 (92.8)	155 (94.5)		
Former smoker	6 (1.8)	3 (1.8)		
Fewer than 15 cigarettes/day	15 (4.5)	5 (3.0)		
15 cigarettes or more/day	3 (0.9)	1 (0.6)		
Rather not answer	4 (—)	1 (—)		
Hypertension			0.497^b^	0.076
No	296 (88.1)	146 (88.5)		
Did not know	19 (5.7)	6 (3.6)		
Yes, not medicated	5 (1.5)	1 (0.6)		
Yes, medicated	16 (4.8)	12 (7.3)		
Thrombophilia			0.138^b^	0.085
No	265 (78.9)	137 (83.0)		
Did not know	69 (20.5)	25 (15.2)		
Yes	2 (0.6)	3 (1.8)		

Abbreviations: BMI, body mass index; ES, effect size; —, Not calculated.

^a^ At least 150 min of moderate aerobic activity per week or at least 75 min of vigorous aerobic activity per week.

^b^ *χ* ^2^‐test of independence.

^c^ Fisher's exact test.

^d^ Indicates cells in which the expected values differ statistically from the observed values.

**TABLE 4:** Binary logistic regression model with formulation of contraceptive as the dependent variable and age (in years) as the control variable of women using hormonal contraception in Rio de Janeiro.

Independent variable	OR	CI 95%	*P*
BMI			
Underweight	—	—	
Healthy weight	0.598	0.131; 1.989	0.424
Overweight	1.395	0.881; 2.205	0.155
Obesity	0.855	0.473; 1.516	0.595
Diabetes			
No	—	—	
Did not know	1.167	0.306; 4.107	0.813
Yes	1.716	0.364; 7.869	0.482
Sedentarism			
No	—	—	
Yes	1.294	0.859; 1.957	0.217
High cholesterol			
No	—	—	
Did not know	0.524	0.206; 1.211	0.134
Yes. not medicated	0.460	0.220; 0.895	0.022
Yes. medicated	0.934	0.293; 2.843	0.904
Smoking			
No	—	—	
Former smoker	1.058	0.203; 4.678	0.942
Fewer than 15 cigarettes/day	0.683	0.208; 1.928	0.484
15 cigarettes or more/day	0.457	0.020; 4.330	0.516
Hypertension			
No	—	—	
Did not know	0.751	0.237; 2.129	0.599
Yes. not medicated	0.537	0.027; 3.809	0.567
Yes. medicated	1.033	0.406; 2.586	0.944
Thrombophilia	—	—	
No	0.726	0.418; 1.228	0.235
Did not know	1.903	0.277; 15.772	0.506

*Note*: Reference category for type of contraceptive: “combined contraceptive.” Nagelkerke pseudo *R* ^2^ = 0.138. *χ* ^2^(18) = 51.821; *P* < 0.001.

Abbreviations: BMI, body mass index; CI, confidence interval; OR, odds ratio; —, Not calculated

Of the women in the study, 91.06% reported using the contraceptive with a prescription, with 436 prescribed by a doctor and 20 prescribed by another healthcare professional. Surprisingly, 83.33% (30 out of 36 women) of participants in category 3 and 100% (three women) of participants in category 4 reported using the contraceptives with a prescription. There was no correlation between self‐medication or prescription and WHO criteria (*χ* ^2^(3) = 1.651; *P* = 0.648) (Table 5).

**TABLE 5:** Contraceptive use according to prescription and WHO categories in Rio de Janeiro.

WHO category	Prescription number (%)	Self‐medication number (%)	Total number (%)
1	24 (92.31)	2 (7.69)	26 (100)
2	130 (88.44)	17 (11.56)	147 (100)
3	30 (83.33)	6 (16.67)	36 (100)
4	3 (100)	0 (0)	3 (100)

*Note*: World Health Organization (WHO) categories for contraceptive eligibility: (1) a condition for which there is no restriction for the use of the contraceptive method; (2) a condition where the advantages of using the method generally outweigh the theoretical or proven risks; (3) a condition where the theoretical or proven risks usually outweigh the advantages of using the method; and (4) a condition that represents an unacceptable health risk if the contraceptive method is used. Statistical analysis by *χ* ^2^ independence test. *P* > 0.05.

We also investigated whether healthcare professionals asked them about CV conditions included in MEC. Of participants, 33.73% (169) responded that no question was posed to them. More than half of smokers (52.27%), women with a family history of CVD (51.72%), and those with actual CVD (54.73%) reported not being asked about it. Moreover, 49.17%, 35.13%, and 19.96% of women with hypertension, high cholesterol, or thrombophilia, respectively, reported they were not asked about it.

**FIGURE 2:**
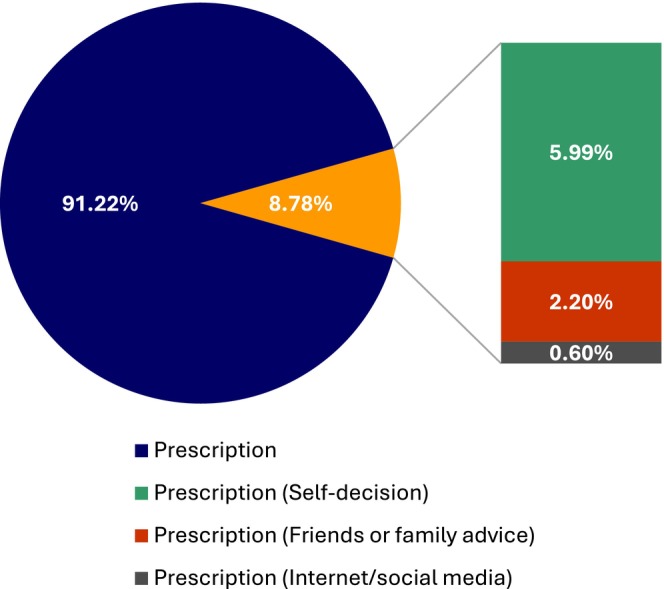
The main information sources for hormonal contraception self‐medication in Rio de Janeiro.

Among self‐medicated women (8.78%, 44 women), most of them do not have CV risk factors included in MEC, 12 women are in category 1 or 2, and six are in category 3. Thus, six women present CV risk factors that could outweigh the advantages of the contraceptive chosen. Self‐decision (30 women), advice from friends or family (11 women), and information on the internet/social media (three women) were the primary sources of information for self‐medication (Figure 2). There was no correlation between the use of combined or progestin‐only contraceptives and self‐medication (*χ* ^2^(1) = 0.146; *P* = 0.703).

## 4 DISCUSSION

In this study, we evaluated the use of hormonal contraceptive methods according to the CV profile of women in Rio de Janeiro state, the third most populous and the second largest economy in Brazil. Sex‐specific differences play an important role in CV health and in the development of diseases, where combined contraceptives are associated with an increased CV risk.^10^ This is of particular importance because CVD is the leading cause of death in women,^12^ and hormonal contraceptives are an important method of contraception worldwide,^1^ a scenario that is also found in Southeast Brazil.^15^, ^18^ Thus, it is important to understand the CV risk profile of users and the types of hormonal contraceptives most used to improve women's health care.

Regarding CV profile, only 6.37% of participants reported actual CVD, and most women have at least one CV risk factor, mainly sedentarism, overweight, and obesity, which were also identified as prevalent in Rio de Janeiro by the Surveillance System of Risk and Protection Factors for Chronic Diseases by Telephone Survey.^19^ However, in our sample, 55.18%, 29.48%, and 19.72% of women were sedentary, with overweight and obese, respectively, compared to 73.4%, 60.1%, and 25.5% found in the VIGITEL Survey.^19^ The same pattern was observed for other CV risk factors, such as hypertension, diabetes, and smoking, which were less frequent in our study. This discrepancy might be due to differences in the educational level of the participants in the two surveys because education is one of the determinants of CV health.^20^ In our study, 44.42% of women have a university education. This percentage is much higher than what was found in the Brazilian population^21^ and Rio de Janeiro state.^19^ In addition, the participants of the study needed to have internet access to participate in the survey, which might indicate a higher level of education and a higher economic status than the overall population of Rio de Janeiro.

The results of our study concur with the data found in the United Nations World Family Planning 2022, which shows that oral contraceptives were the most used contraceptive method in Brazil in 2020. Lago et al. (2020) also found that oral contraceptives were the most frequent contraceptive method used by women in São Paulo city in 2015.^22^ Among oral contraceptives, COC was the main contraceptive choice of the participants in our study, which was also found to be the preferred method of Brazilian women.^23^ Thus, our data and other studies indicate that short‐acting methods are preferred by women in Brazil.

The pattern of contraceptive use varied according to women's age. We found a higher frequency of use of POC for women older than 40 years, which was expected because CV risk factors are more prevalent in this group and might represent contraindications for COC use.^10^ For example, thromboembolism risk increases with age.^24^, ^25^

Marital status followed the same pattern, with married/in common law married and widowed women using POC more frequently, a finding that correlates with age. In contrast, single women use combined contraceptives more frequently. Marital status can be considered an important factor as married women are more prone to change their contraceptive method than single women.^26^ It was shown that in 2020, 820 million married women of reproductive age used contraceptive methods, where 114 million (14.1%) used oral contraceptives and 20 million (2.4%) used subdermal implants. In contrast, of 146 million unmarried women, 36 million (25%) used oral contraceptives and 5 million (3.8%) used subdermal implants.^1^

In the context of contraceptive usage and MEC, most women used the contraceptive recommended for their health condition and reported using it with a prescription. Although physical inactivity and being overweight are recognized as factors that increase CV risk,^27^ they are not included in MEC.^10^ Obesity is not a contraindication for contraceptive usage, as it is included in categories 1 or 2 of MEC depending on the type of contraceptive. However, there is evidence that the use of COC by obese women significantly increases the risk of venous thromboembolism, indicating that POC should be recommended for this group. It is estimated that obese women have a 12–14 times higher risk of venous thromboembolism when using COC than non‐obese women.^9^

Hyperlipidemia is categorized as 2 for POC but as 2/3 for combined contraceptives. In this scenario, categorization is determined by the severity of hyperlipidemia and the existence of other CV risk factors. COC's effect on lipid metabolism is determined by its estrogen and progestogen components, with estrogen increasing HDL and triglyceride levels and progestogens, particularly androgenic ones, decreasing HDL and increasing LDL levels.^28^ Further, hyperlipidemia could increase the risk of arterial thromboembolism in COC users.^29^ In our study, we found that women who reported having treated hyperlipidemia used POC less frequently, indicating a potential gap in clinical management or contraceptive guidance in CV risk scenarios.

Combined oral contraceptives was the contraceptive type mostly associated with contraindicated use, even among women with prescriptions. This might have happened because, according to most women's reports, a higher number of health professionals did not ask about their health condition. In the same way, CVD is underestimated, underdiagnosed, and less investigated in women.^12^ Further, informed decision‐making is one of the human rights principles that guide family planning and involves explaining the side effects and risks associated with the contraceptive method.^27^

We found that most hypertensive women use COC, which can be categorized as 3 or 4. COC use in hypertensive women is well known to raise CV risk, an effect linked to the estrogen component. Additionally, the CV risk can be raised by the existence of additional risk factors, which must be examined simultaneously.^30^ A previous study found that in Brazil, hypertension was the most common contraindication among COC users,^16^ indicating that it appears to be an overlooked risk factor.

Two women reported using COC despite having thrombophilia, which is an important contraindication for this type of contraceptive due to the increased risk of venous thromboembolism.^31^ The risk of venous thromboembolism changes with the duration of use, estrogen dose, and progestogen type. It is high in the first year of usage, in formulations with a high ethinyl estradiol dose, and in COC containing drospirenone, gestodene, desogestrel, and cyproterone.^10^, ^32^ Although the venous thromboembolism risk stabilizes after the first year of use, and both women reported using the COC for more than 10 years, they both used contraceptives containing progestogens associated with a higher risk of venous thromboembolism (drospirenone and cyproterone). This highlights the need to identify women at increased risk of venous thromboembolism when prescribing the hormonal contraceptive method.^31^

Online surveys have demonstrated effectiveness as a methodological strategy for data collection since they enable the elimination of geographical barriers and access to a diverse population. Further, digital platforms allow more efficient and secure data storage, management, and analysis. In contrast, online surveys have limitations such as sampling and response bias and question misunderstanding.^33^ Although the study design was carefully considered for the collection of relevant and meaningful data from the investigated population, sampling bias was a limitation of our study, as almost half our sample has a high level of education. Thus, the problem related to the use of hormonal contraceptives, mainly COC, by women with CV risk might have been underestimated, and future research is needed to deepen our findings.

## 5 CONCLUSION

In conclusion, our findings show that hormonal contraception is an important component of family planning in Rio de Janeiro state and provide valuable contributions to the understanding of the CV risk profile of women. Although most women use the hormonal contraceptive that is appropriate to their CV profile, our results show that some women use contraindicated contraceptives, even with a medical prescription.

Therefore, WHO MEC should be routinely used for hormonal contraception prescriptions to promote the safe and individualized use of contraceptive methods. Improved clinical management and CV risk assessment for women protects their health and ensures an evidence‐based family planning strategy.

## AUTHOR CONTRIBUTIONS

**Isabela Pereira Fonseca Brusth:** Methodology, Formal analysis, Investigation, Writing—Original Draft. **Juliana Montani Raimundo:** Conceptualization, Methodology, Formal analysis, Investigation, Resources, Writing—Review & Editing, Supervision, Project administration. **Helene Nara Henriques Blanc:** Conceptualization, Methodology, Formal analysis, Investigation, Resources, Writing—Review & Editing, Supervision, Project administration.

## FUNDING INFORMATION

This work was supported by the FAPERJ—Fundação Carlos Chagas de Amparo à Pesquisa do Estado do Rio de Janeiro (E‐26/203.043/2019).

## CONFLICT OF INTEREST STATEMENT

The authors have no conflicts of interest to declare.

## Data Availability

Data available upon request from the journal.
